# Nutrition in Skin Picking Disorder and Trichotillomania

**DOI:** 10.3389/fpsyt.2021.761321

**Published:** 2021-11-23

**Authors:** Jon E. Grant, Stephanie Valle, Samuel R. Chamberlain

**Affiliations:** ^1^Department of Psychiatry and Behavioral Neuroscience, University of Chicago, Chicago, IL, United States; ^2^Department of Psychiatry, Faculty of Medicine, University of Southampton, Southampton, United Kingdom; ^3^Southern Health NHS Foundation Trust, Southampton, United Kingdom

**Keywords:** nutrition, impulsivity, compulsivity, trichotillomania, skin picking disorder

## Abstract

**Objective:** Excessive calorie intake constitutes a global public health concern, due to its associated range of untoward outcomes. Impulsivity and compulsivity have been linked to dietary intake. However, nothing is known about dietary intake and body-focused repetitive behaviors, despite their classification as obsessive-compulsive related conditions, and high co-morbidity with impulsive and compulsive conditions.

**Methods:** One hundred and ninety six adults with trichotillomania or skin picking disorder were recruited. Dietary intake over the preceding year was quantified using the Dietary Fat and Free Sugar Short questionnaire. Relationships between dietary fat/sugar intake and behaviors were evaluated using regression modeling.

**Results:** Sugar intake was significantly related to higher trans-diagnostic compulsivity (*p* = 0.011) and higher non-planning impulsivity (*p* = 0.013) In terms of saturated fat intake, there was no significant relationship to the explanatory variables. A combination high fat/high sugar diet was significantly associated with higher motor impulsivity (*p* = 0.005).

**Conclusions:** Past-year nutrition appears to be significantly associated with trans-diagnostic impulsivity and compulsivity. The role of poor nutrition in these disorders and related conditions, and its link with impulsivity and compulsivity, requires longitudinal research attention; and clinical work should address not only psychiatric symptoms but also impact of lifestyle of overall health.

## Introduction

Body-focused repetitive behaviors (BFRBs) are a group of related psychiatric disorders that includes trichotillomania and skin picking disorder. These behaviors underpin complex disorders that cause people to repeatedly touch their hair and body in ways that result in physical damage. Both of these disorders generally start in childhood or late adolescence and are associated with distress and reduced quality of life (1–3). Although the etiology of BFRBs is unknown, people have wondered about the possible role of diet and nutrition in the genesis or exacerbation of these behaviors.

The role of diet may be particularly relevant to BFRBs given that two possible treatment options have suggested dietary imbalances in these disorders. First, N-acetyl cysteine, a proteinogenic amino acid in high-protein foods, has shown benefit in reducing hair pulling and skin picking in two double-blind studies (4, 5). Second, although unsuccessful in a double-blind study of trichotillomania, inositol, an endogenous isomer of glucose found in fruits, grains, nuts and beans, has been used by many people with BFRBs with some anecdotal reported benefits (6). Furthermore, evidence to date has suggested that dietary changes (e.g., eating a gluten free diet or increasing vitamin D and B12) may play a role in reducing the symptoms of Tourette's and obsessive compulsive disorder, disorders with some phenomenological and possibly genetic relationship to trichotillomania and skin picking disorder (7, 8). Given the provocative suggestion of the role of diet in mental health disorders, the recent advice by the Royal College of Psychiatrists in the UK, regarding the importance of eating well for those with mental health issues (https://www.rcpsych.ac.uk/mental-health/problems-disorders/eating-well-and-mental-health), is perhaps all the more prescient. Although sound advice, there has been no research regarding the diets of people with BFRBs. Thus, until more is understood about their diets, recommendations regarding improved eating habits in people with BFRBs are difficult to make.

The role of diet in BFRBs also merits scrutiny due to links in other literature between dietary intake and impulsivity (i.e., behaviors or actions that are inappropriate, premature, unduly thought out, and risky, leading to untoward outcomes) and compulsivity (i.e., broadly defined as a tendency toward repetitive, habitual actions, which an individual feels compelled to perform). For example, added sugar in a person's diet has been associated with impulsive choices (9), and vitamin deficiencies of E, C, and B12 have been associated with obsessive-compulsive disorder (10). Poor diet (e.g., sugar-sweetened beverages, processed foods) coupled with loss of control over eating (e.g., over-eating) have been linked, to a variety of impulsive/compulsive symptoms, such as attention-deficit hyperactivity disorder (ADHD) and (relatedly) binge-eating (11–13). BFRBs have impulsive/compulsive features, such as impaired response inhibition and ritualized behaviors, and comorbidities such as obsessive-compulsive disorder and behavioral addictions (14–16).

In view of the evident paucity of data examining diet in those with trichotillomania and skin picking disorder, and potential relationships with trait impulsivity/compulsivity, the current study sought to examine the nutrition of adults with BFRBs and the relationship of their nutrition to a range of domains, problematic behaviors, and mental health issues. Based on the literature that has found associations between high sugar diets and impulsivity, we hypothesized that high calorie intake in the form of increased sugars would be associated with more severe pulling and picking; as well as with higher trans-diagnostic impulsivity and compulsivity.

## Materials and Methods

One hundred and ninety six participants were recruited from the general community via online advertisements for a study on nutrition in trichotillomania and skin picking disorder. Inclusion criteria were the following: aged 18–65 years; current diagnosis of a body-focused repetitive behavior (i.e., trichotillomania or skin picking disorder) based on DSM-5 criteria; active pulling or picking behavior at time of enrollment; and ability to provide active consent for participation. Subjects were excluded if they were unable to give informed consent or were unable understand/undertake the study procedures; reported infrequent incidence of target behavior (i.e., <1 time per week) that does not meet DSM-5 (or standard measure) criteria for a body-focused repetitive behavior; reported unstable use of psychotropic medication (i.e., stability for ≥ 3 months has not been met); and/or had a lifetime history of bipolar disorder or any psychotic disorder or a current (past 3-month) substance use disorder.

The study consisted of a single face-to-face assessment via an online video platform, coupled with completion of an online survey. During the consent processes, each participant read an oral consent script or they were given a paper consent form at their visit. A copy of this script was also placed in the packet of surveys that they completed. A refusal to respond or inaction was taken as a denial of consent. It was made clear to each participant that they were free to withdraw from the study at any time if they wished to revoke their consent. After completing consent procedures, subjects were provided with copies of the surveys. Instructions were given on how to complete the forms, as well as a reiteration that they were free to revoke their consent at any time, including skipping questions that they do not feel comfortable answering.

All study procedures were carried out in accordance with the Declaration of Helsinki. The Institutional Review Board of the University of Chicago approved the study and the consent statement. Participants were compensated with a $15 gift card for a local department store.

### Assessments

Participants were asked to complete standard diagnostic interviews and basic demographic information. In addition, participants completed the following measures:

*The Dietary Fat and Free Sugar Short questionnaire (DFS)* was used to quantify intake of saturated fat and free sugars over the preceding year (17). The DFS is a valid and reliable 26-item self-report scale that assesses macronutrient intake over the year prior to the assessment. The scale focuses on the intake of saturated fat and simple sugars and provides separate scores for these nutrients to distinguish individuals with a high intake of both nutrients, a high intake of only one nutrient, or a low intake of both. Thus, there is a saturated fat subscale, a sugar subscale, and a fat-sugar subscale. Prior validation indicates that each score be considered in its own right (17).

#### Massachusetts General Hospital Hair Pulling Scale (MGH-HPS)

The MGH-HPS is a 7-item, self-report scale that rates urges to pull hair, actual amount of pulling, perceived control over behavior, and distress associated with hair pulling over the past 7 days (18).

#### Skin-Picking Scale—Revised (SPS-R)

The SPS-R is a 8-item, reliable, valid, self-rated scale assessing picking urges, thoughts, and behaviors during the previous 7 days. Each item is rated 0 to 4 with a possible total score of 32 (19).

#### Barratt Impulsiveness Scale (BIS)

The BIS is composed of 30 items describing common impulsive behaviors and preferences (20). The BIS is a self-report measure assessing attentional, motor, and non-planning dimensions of impulsivity. The measure consists of 30 questions, with each rated on a scale of 1 (“Rarely/Never”) to 4 (“Almost Always/Always”).

#### Sheehan Disability Scale (SDS)

The SDS is a self-report tool developed to assess functional impairment in the following domains: work/school, social life, and family life. Impairment in each area is rated on a 10-point visual analog scale (21).

#### Cambridge-Chicago Trait Scale (CHI-T)

The CHI-T is a 15-item scale measuring trans-diagnostic compulsivity. Rated on a 4-point scale: strongly disagree—strongly agree. Higher scores indicate higher levels of compulsivity (22, 23).

### Statistical Analysis

In order to assess relationships between variables of interest and dietary intake scores, three separate standard least squares regression models were conducted using JMP Pro Software. Explanatory variables in each model comprised: age, symptom severity (MGH, SPS), disability (SDS), impulsivity (BIS motor, attentional, and non-planning), and compulsivity (CHI-T). Outcome variables were the dietary intake subtotal scores. Regression models were described in terms of Root Mean Square Error (RMSE, which can be interpreted as standard deviation of unexplained variance, lower is better fit), *R*^2^ (proportion of variance explained by the model), and overall *p* statistic (test as to whether the overall model statistically explains more variance than the null model). Statistical significance for the overall models was defined as *p* < 0.05 corrected, two-tailed.

## Results

The participant sample consisted of 196 adults (mean age = 33.36; 186 [94.9%] female), of whom 47 (23.98%) had a primary trichotillomania diagnosis, 83 (42.35%) had skin picking disorder, and 66 (33.67%) had both trichotillomania and skin picking disorder. Table 1 presents demographic and clinical characteristics of the participants. Correlations between continuous predictor variables entered into the models are provided in Supplementary Table 1.

**Table 1:** Demographic and clinical features.

	**Adults with BFRBs**	**Skin picking disorder**	**Trichotillomania**	**Both trichotillomania and skin picking disorder**
	**(*N* = 196)**	**(*N* = 83)**	**(*N* = 47)**	**(*n* = 66)**
Age, years	33.36	33.84	35.19	31.45
Gender, female, *n* (%)	186 (94.9)	81 (97.6)	44 (93.6)	61 (92.4)
Racial-ethnic group, White Caucasian, *n* (%)	163 (83.2)	75 (90.4)	37 (78.7)	51 (77.37)
**Current relationship status**, ***n*** **(%)**				
Single Married Divorced Widowed	94 (48.0) 93 (47.5) 7 (3.6) 2 (1.0)	40 (48.2) 41 (49.4) 0 (0) 2 (2.4)	20 (42.6) 25 (53.2) 2 (4.3) 0 (0)	34 (51.5) 27 (40.9) 5 (7.6) 0 (0)
**Education level**, ***n*** **(%)**				
High school of less At least some college or more	0 (0) 196 (100)	0 (0) 83 (100)	0 (0) 47 (100)	0 (0) 66 (100)
**Any current psychiatric comorbidity**, ***n*** **(%)**				
ADHD Alcohol/substance use disorder Major depressive disorder Obsessive Compulsive disorder Any anxiety disorder	36 (18.4) 8 (4.1) 117 (59.7) 63 (32.1) 124 (63.3)	12 (14.5) 2 (2.4) 50 (60.2) 24 (28.9) 51 (61.4)	9 (19.1) 0 (0) 21 (44.7) 15 (31.9) 24 (51.1)	15 (22.7) 6 (9.1) 46 (69.7) 24 (36.4) 49 (74.2)
**The Dietary Fat and Free Sugar Short questionnaire (DFS)**				
Saturated Fat subscale (SD)	27.20 (6.15)	27.38 (7.09)	26.51 (5.47)	27.48 (5.34)
Sugar subscale (SD)	8.77 (3.64)	8.60 (3.69)	8.39 (3.28)	9.27 (3.82)
Fat-Sugar subscale (SD)	16.02 (4.49)	15.80 (4.93)	15.68 (3.56)	16.53 (4.55)
MGH-HPS total score (SD)	10.45 (8.94)	0.75 (2.58)	16.43 (4.91)	16.92 (5.41)
SPS-R total score (SD)	12.19 (7.99)	17.22 (5.02)	1.23 (2.26)	13.68 (5.88)
SDS total score (SD)	11.92 (8.41)	11.55 (8.16)	11.06 (8.68)	13 (8.55)
BIS-11 total score (SD)	65.56 (11.69)	64.80 (10.95)	65.13 (12.26)	66.83 (12.25)
CHI-T total score (SD)	26.03 (7.48)	26.38 (8.79)	24.26 (6.50)	26.81 (6.25)

*All scores are mean scores unless stated otherwise; abbreviations: MGH-HPS, Massachusetts General Hospital Hair Pulling Scale; SPS-R, Skin Picking Scale Revised; SDS, Sheehan Disability Scale; BIS, Barratt Impulsiveness Scale; CHI-T, Cambridge-Chicago Trait Scale*.

In terms of saturated fat intake, there was no significant relationship to the explanatory variables (RMSE = 6.108, *R*^2^ = 0.05, *p* = 0.401).

High sugar intake was significantly related to the explanatory variables overall (RMSE 3.38, *r*^2^ = 0.18, *p* < 0.001). As shown in Table 2, this was due to higher compulsivity and higher non-planning impulsivity being associated with higher sugar intake, in the people with BFRBs.

**Table 2:** Regression model results, linking explanatory variables to sugar intake.

**Term**	**Std Beta**	**Design Std Error**	**Estimate**	**Std Error**	**t Ratio**	**Prob>|t|**
Intercept	0	0.60	−0.72	2.05	−0.35	0.7262
Age	−0.02	0.01	−0.01	0.02	−0.29	0.7745
MGH Total	0.02	0.01	0.01	0.03	0.27	0.7847
SPS Total	−0.05	0.01	−0.02	0.04	−0.58	0.5634
SDS Total	0.03	0.01	0.01	0.04	0.37	0.7098
Attentional impulsivity	0.04	0.02	0.03	0.07	0.41	0.6797
Motor impulsivity	0.10	0.03	0.10	0.09	1.08	0.2833
Non-planning impulsivity	0.27	0.02	0.19	0.07	2.52	0.0127^*^
CHI-T Total	0.23	0.01	0.11	0.04	2.58	0.0109^*^

* *p < 0.05*.

High fat-sugar was significantly related to the explanatory variables overall (RMSE 4.25, *R*^2^ = 0.12, *p* = 0.005). As shown in Table 3, this was due to higher fat-sugar intake being associated with higher motor impulsivity.

**Table 3:** Regression model results, linking explanatory variables to fat-sugar intake.

**Term**	**Std Beta**	**Design Std Error**	**Estimate**	**Std Error**	***t* Ratio**	**Prob>|t|**
Intercept	0	0.60	6.74	2.56	2.63	0.0094^*^
Age	0.06	0.01	0.02	0.03	0.80	0.4235
MGH total	−0.03	0.01	−0.01	0.04	−0.35	0.7291
SPS total	0.02	0.01	0.01	0.05	0.16	0.8713
SDS total	−0.01	0.01	−0.00	0.05	−0.08	0.9360
Attentional impulsivity	0.16	0.02	0.17	0.09	1.87	0.0630
Motor impulsivity	0.28	0.03	0.34	0.12	2.85	0.0050^*^
Non-planning impulsivity	−0.12	0.02	−0.10	0.09	−1.09	0.2782
CHI-T Total	0.11	0.012	0.06	0.05	1.18	0.2406

* *p < 0.05*.

## Discussion

This study, the first examination of nutrition in BFRBs, found that high sugar intake was associated with greater compulsivity and non-planning impulsivity, whereas a diet high in both saturated fat and sugar was associated with greater motor impulsivity. Diet however was not associated with severity of either trichotillomania or skin picking. What then do we make of these findings for people with BFRBs?

BFRBs have often been characterized as having both an impulsive aspect to them [for example, elevated stop signal reaction time (16)] as well as a compulsive element [for example, anxiety reduction by repetitive behaviors, (14)]. Interestingly, high sugar diet was associated with elevated aspects of both compulsivity and one feature of impulsivity (non-planning) and high fat-sugar diet was associated with a different feature of impulsivity (motor impulsivity). Overall, the findings suggest that trans-diagnostic impulsivity and compulsivity are associated with aspects of past-year dietary intake in people with BFRBs, but do not appear to contribute to past-week variation in BFRB symptom severity itself.

There are several possible explanations for these findings. First, diet may be a variable in the initiation of pulling and picking via the domains of impulsivity and compulsivity but may have little influence on the day to day fluctuations in pulling and picking severity. If true, then diet could be a focus for primary interventions primarily in those young people vulnerable to developing a BFRB. Second, diet was measured for the past year, impulsivity and compulsivity were assessed as trait measures, and severity of symptoms were measured for the week before study entry. Thus, diet may influence impulsivity and compulsivity, and in addition be a variable in worsening pulling or picking behavior, but our current measures of severity are not assessing the longer time-line that diet would affect. Third, it could be that impulsivity and compulsivity are affecting diet and hair pulling or skin picking. For example, a participant's impulsivity results in a high sugar or high sugar/high fat diet and results in the decision to pull or pick. Finally, it could be that diet increases impulsivity and compulsivity in those with BFRBs but that severity of behaviors is being driven by other variables not measured in this study.

What are the clinical implications of these new findings? Given that dietary intake related to aspects of impulsivity/compulsivity, which themselves are implicated across a range of conditions, this draws attention to the need for holistic care focusing not only on cardinal symptoms but also associated lifestyle factors, which may worsen long-term outcomes or quality of life for patients with BFRBs and other related conditions. The link between dietary intake and trans-diagnostic compulsivity merits longitudinal research, Theoretically, poor nutrition could affect neurodevelopment, lead to impulsive and compulsive traits, in turn predisposing to a range of symptoms. One could hypothesize that reducing sugar and saturated fats in people with BFRBs might possibly reduce both impulsivity and compulsivity, but to what extent and to what end remains unknown.

There are several limitations that should be considered in relation to this study. The study did not collect information on medication or history of psychotherapy both of which could have affected BFRB severity. This was cross-sectional and no causality can be inferred. The links between impulsivity, compulsivity, and calorie intake differed depending on the particular sub-score from the dietary instrument, and follow-up work is needed to explore why this might have been the case. We looked at dietary intake in people with BFRBs, rather than using a case-control design. Finally, the study was not designed to characterize psychometric properties of the scales, since this has been done in previous work.

In conclusion, we found that aspects of past-year dietary intake were significantly associated with trans-diagnostic impulsivity and compulsivity, highlighting the need to address lifestyle factors in clinical settings, as well as the research need for longitudinal studies exploring brain development, traits, and ultimate symptom manifestation for BFRBs and other conditions. At the same time, we did not find evidence that past-year dietary intake relates significantly to core symptom severity of picking or pulling.

## Data Availability Statement

The datasets presented in this article are not readily available because data use sharing agreements would be necessary. Requests to access the datasets should be directed to jongrant@uchicago.edu.

## Ethics Statement

The studies involving human participants were reviewed and approved by University of Chicago. The patients/participants provided their written informed consent to participate in this study.

## Author Contributions

All authors contributed equally to the conceptualization, analysis, and drafting of the research report.

## Conflict of Interest

JG has received research grants from the Otsuka, Biohaven, Promentis, and Avanir Pharmaceuticals. He receives yearly compensation for acting as editor-in-chief of the Journal of Gambling Studies and has received royalties from Oxford University Press, American Psychiatric Publishing, Inc., Norton Press, and McGraw Hill. SC receives honoraria from Elsevier for editorial work at Comprehensive Psychiatry; and Neuroscience and Biobehavioral Reviews. SC previously consulted for Promentis. The remaining author declares that the research was conducted in the absence of any commercial or financial relationships that could be construed as a potential conflict of interest.

## Publisher's Note

All claims expressed in this article are solely those of the authors and do not necessarily represent those of their affiliated organizations, or those of the publisher, the editors and the reviewers. Any product that may be evaluated in this article, or claim that may be made by its manufacturer, is not guaranteed or endorsed by the publisher.
