# Supplementary material for: Nutrition in Skin Picking Disorder and Trichotillomania
Source: Front Psychiatry. 2021 Nov 23;12:761321. doi: 10.3389/fpsyt.2021.761321 (PMC8650212; doi:10.3389/fpsyt.2021.761321)

Supplementary Table 1. Correlation matrix (Pearson’s r) between predictor variables in the sample.


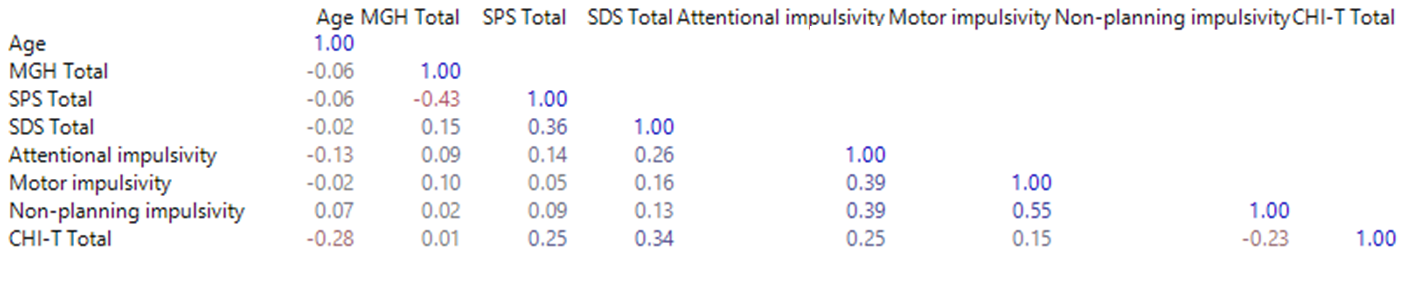


Supplementary Panel 1. Diagnostics to assess model assumptions for examination of sugar intake. Inspection of the plot of predicted versus residuals (top) and quantile plot for residuals (bottom) indicated that model assumptions were reasonably met.


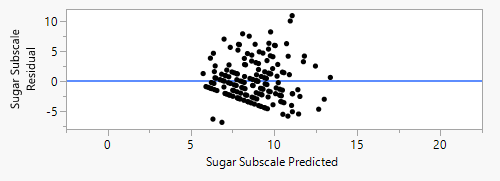


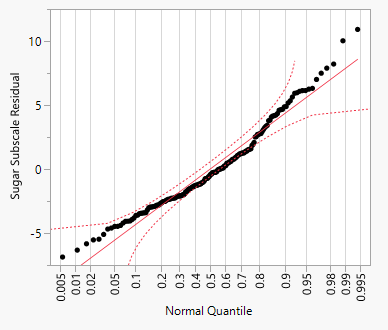


Supplementary Panel 2. Diagnostics to assess model assumptions for fat-sugar intake. Inspection of the plot of predicted versus residuals (top) and quantile plot for residuals (bottom) indicated that model assumptions were reasonably met.


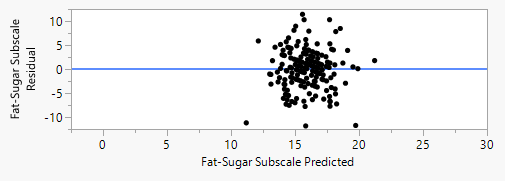


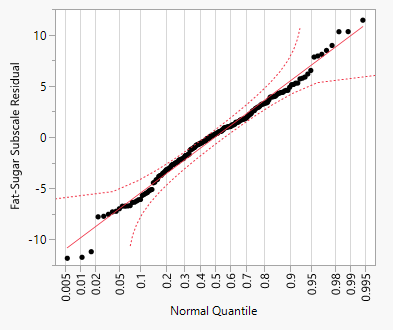

Supplement: Supplementary file 1 [file Table_1.DOCX]
